# Detecting Debonding between Steel Beam and Reinforcing CFRP Plate Using Active Sensing with Removable PZT-Based Transducers

**DOI:** 10.3390/s20010041

**Published:** 2019-12-19

**Authors:** Jian Jiang, Jinwei Jiang, Xiaowei Deng, Zifeng Deng

**Affiliations:** 1 Key Laboratory of Earthquake Geodesy, Institute of Seismology, China Earthquake Administration, Wuhan 430071, China; jjiang19@uh.edu; 2 Wuhan Institute of Earthquake Engineering Co., Ltd., Wuhan 430071, China; 3 Department of Mechanical Engineering, University of Houston, Houston, TX 77204, USA; ctdxw@mail.scut.edu.cn (X.D.); zifengdeng_scut@163.com (Z.D.); 4 School of Civil Engineering and Transportation, South China University of Technology, Guangzhou 510641, China

**Keywords:** carbon fiber reinforced polymer (CFRP), steel structure retrofit or reinforcement by CFRP, lead zirconate titanate (PZT), debonding detection, active sensing

## Abstract

Carbon fiber reinforced polymer (CFRP) plates are widely used to retrofit or reinforce steel structures, and the debonding damage between the steel structure and the CFRP plate is a typical failure in strengthening steel structures. This paper proposes a new approach to detecting debonding between a steel beam and a reinforcing CFRP plate by using removable lead zirconate titanate (PZT)-based transducers and active sensing. The removable PZT-based transducers are used to implement the active sensing approach, in which one transducer, as an actuator, is used to generate stress wave, and another transducer, as a sensor, is used to detect the stress wave that propagates across the bonding between the steel beam and the reinforcing CFRP plate. The bonding condition significantly influences the received sensor signal, and a wavelet-packet-based energy index (WPEI) is used to quantify the energy of the received signal to evaluate the severity of debonding between the steel beam and the reinforcing CFRP plate. To validate the proposed approach, experimental studies were performed, and two removable PZT-based transducers were designed and fabricated to detect the debonding between a steel beam and the reinforcing CRFP plate. The experimental results demonstrate the feasibility of the proposed method in detecting the debonding between a steel beam and the reinforcing CFRP plate using removable PZT-based transducers.

## 1. Introduction

Steel structures of various forms are widely used in constructions [1,2], however, with adverse factors such as corrosion [3,4], dynamic loads [5,6], and impacts [7,8], steel structures are subject to damages [9,10]. Carbon fiber reinforced polymer (CFRP) materials are widely used to retrofit or reinforce damaged steel structures [11,12,13] due to their high strength-to-weight ratio, excellent corrosion resistance, and fast and easy installation. More recently, research on the application of CFRP materials for reinforcement of steel structures has received much attention [14]. CFRP has been demonstrated as promising for strengthening steel structures [15], especially for the bending components, which can be strengthened conveniently by bonding a CFRP sheet or plate to the tension face [16].

Among the CFRP materials used for reinforcement of steel structures, CFRP sheets and CFRP plates are most commonly used. Meanwhile, compared with CFRP sheets, CFRP plates possess more advantages for strengthening damaged flexural components [17]. Therefore, the application of CFRP plates for strengthening steel structures has attracted much attention. Deng et al. [18] conducted an experimental and theoretical study on notched steel beams strengthened by bonding CFRP plates. Hosseini et al. [19] introduced a prestressed unbonded reinforcement (PUR) system to strengthen existing fatigued steel members, and they also studied bond behavior and anchorage resistance of prestressed CFRP plates to steel substrate. Chen et al. [20] investigated fatigue improvements of CFRP-plate-strengthened steel beams. Martinelli et al. [21] investigated the bond behavior of CFRP plates epoxied to the steel substrate.

Studies show that the debonding between a steel structure and the reinforcing CFRP plate is one of the main failure modes [22], which can affect the reinforcement effectiveness [23] and even cause brittleness and sudden failure of strengthened structures [24]. The debonding defects mainly occur in four areas [25]: the CFRP plate, the steel/epoxy interface, the CFRP/epoxy interface, and the epoxy layer. The debonding damage is a typical failure in strengthening steel structures, urgently calling for an effective and nondestructive detection technique, which can help to detect damage timely so that effective measures can be taken to avoid serious consequences. Nondestructive detection techniques, such as acoustic emission technology [26], ultrasonic inspection technology [27], fiber optic sensing [28,29] and X-ray inspection [30], have been applied in structural damage detection. However, most of these conventional methods require complex equipment and algorithms, which may be difficult to deploy in some engineering applications. Therefore, a simple and effective nondestructive testing (NDT) method that can be applied in debonding detection between a steel structure and the reinforcing CFRP plate is necessary.

As a commonly used piezoceramic material, lead zirconate titanate (PZT) has been applied in structural health monitoring [31,32,33] due to its strong piezoelectric effect [34,35] and wide bandwidth [36,37]. With both sensing and actuation functions, PZTs are often used in active sensing methods for structural health monitoring (SHM) and damage detection. For example, the active sensing method was used in characterizing concrete hydration [38], monitoring circular reinforced concrete columns under seismic excitations [39], and detecting damages in circular RC columns [40]. With the PZT transducer, the electro-mechanical impedance (EMI)-based technique was applied to health-monitoring plate-like structures [41], pin connection loosening [42], and grout compactness of concrete-filled steel tubes [43]. There are other applications of PZT transducers in SHM [44,45].

As the propagation of stress wave across the bonding interface is sensitively correlated to the bonding condition, the PZT-enabled active sensing technique is also used in debonding detection and monitoring. Qin et al. [46] studied the bond-slip detection between concrete beams and a steel plate using piezoceramic smart aggregates. Zeng et al. [47] performed bond-slip detection between concrete and steel using shear mode smart aggregates. Xu et al. [48] discovered that the active sensing method can monitor the bond slip between concrete structures and the glass fiber reinforced polymer (GFRP) bars accurately. Di et al. [49] investigated the debonding process between the fiber reinforced polymer (FRP)/steel bars and the hosting structures using PZT probes and acoustic emission. Kong et al. [50] developed an active sensing approach to monitor the cyclic crack of a reinforced concrete column in the process of simulated pseudodynamic loading.

However, few studies on debonding detection between a CFRP plate and steel beam using active sensing have been reported. In addition, the PZT-enabled active sensing methods often require the permanent installation of the transducers on or in the host structures [4,34,38,42,47,48], which brings inconvenience to large-scale implementation. These factors motivate the authors to develop a new approach to detecting debonding between steel structures and the reinforcing CFRP plates. The main innovations of this paper are the development of the removable PZT transducers and the use of them in active sensing to detect the debonding damage between the steel beam and the reinforcing CFRP plate. To verify the feasibility of the proposed PZT-enabled active sensing method, two removable PZT-based transducers were fabricated, and each of them contained a PZT patch, two wires, and a strong magnet. For the testing specimen, a steel beam was bonded with a CFRP plate with epoxy for reinforcement. In this study, three different debonding areas (Area-A, Area-B, Area-C) were preset on the steel beam/CFRP plate interface of the testing specimen, and Area N was set as the healthy status without debonding. The experimental results of the debonding detection clearly show that the magnitudes of received signals decrease significantly from the case without debonding to the case with debonding, and the amount of decrease increases with the severity of the debonding damage, which demonstrates the effectiveness of the debonding detection between steel beam and reinforcing CFRP plate using the removable PZT transducers and the active sensing.

## 2. Principles

### 2.1. Removable PZT-Based Transducer

**Figure 1:**
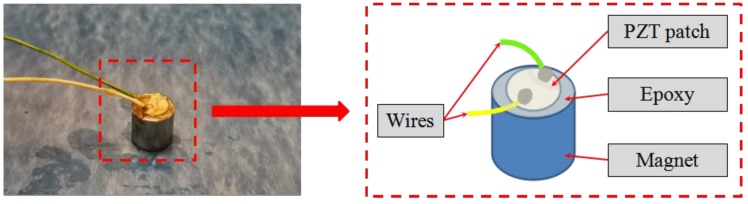
Details of the lead zirconate titanate (PZT) removable transducer.

**Figure 2:**
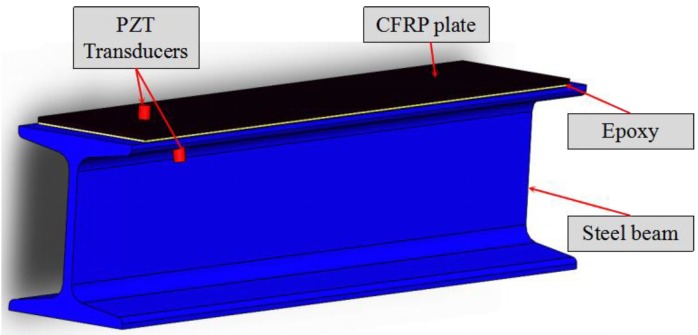
3D sketch of specimen for debonding detection using PZT transducers. CFRP: Carbon fiber reinforced polymer.

Because of the piezoelectricity effect, a PZT patch can be used as either a sensor or an actuator [51], and a PZT patch can generate and detect stress waves [34,42,52]. Traditionally, a PZT patch can be embedded inside a structure or bonded to a structural surface to conveniently enable the active sensing approach [53,54,55]. To detect debonding damages at different locations, a removable PZT transducer was developed in this research. The PZT transducer has a simple design, as shown in Figure 1, and a photo of such a fabricated transducer is also shown in Figure 1. The removable transducer consists of a PZT disk with two wires and a strong cylindrical magnet, and the PZT disk is bonded to the top surface of the magnet by epoxy. Unlike the traditional PZT patch, which can only be used once after being embedded inside or bonded on the structural members, the proposed removable PZT transducer can be easily installed and removed for repeated use. Using the magnet as the carrier of the PZT can exert a required and uniform bonding force between the sensor and the testing surface, which helps to effectively propagate the stress wave. Figure 2 shows the 3D sketch of the specimen for debonding detection. The removable PZT transducers can be easily attached to any steel structure surface. For better propagation of the stress wave, a coupling agent (couplant) on the contact surface was used in the experimental test. These removable PZT transducers have the potential to be integrated with robotic manipulators to automate the debonding detection process.

### 2.2. Piezoceramic-Based Active Sensing Approach

**Figure 3:**
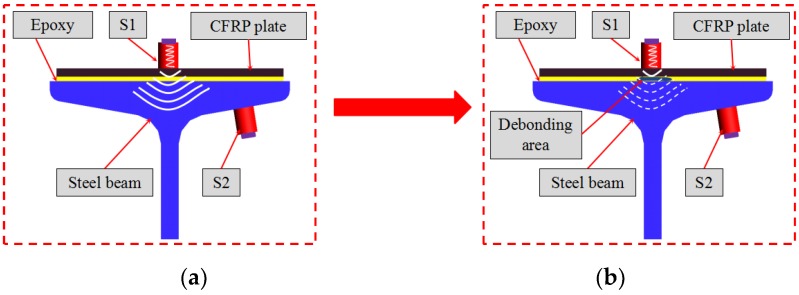
The active sensing schematic in the debonding detection between steel beam and reinforcing carbon fiber reinforced polymer (CFRP) plate: (**a**) received signals of S2 at no debonding area; (**b**) received signals of S2 at debonding area.

Figure 3 shows the active sensing schematic of detecting the debonding damage between steel beam and reinforcing CFRP plate. Two PZT-based removable transducers are used in experimental tests. One transducer is used as an actuator (S1) and the other one is used as a sensor (S2). The stress wave generated by S1 propagates from the magnet to the CFRP plate surface and then propagates to the steel beam through the epoxy layer. In the CFRP-plate-strengthened steel beam, the stress wave propagation from the CFRP plate to the steel beam is sensitively correlated to the bonding condition. When the steel beam and the reinforcing CFRP plate are bonded in a very good condition, the stress wave can effectively propagate to the steel beam through the epoxy easily, as shown in Figure 3a. Otherwise, the received signal of S2 will attenuate rapidly when there is a debond damage in the epoxy layer, as shown in Figure 3b. In addition, the amount of energy decrease is related to the size of the debonding area. A larger debonding area will result in a large energy decrease.

### 2.3. Wavelet-Packet-Based Energy Index

As a quantitative method, the wavelet-packet-based energy index (WPEI) analysis has been used to evaluate the differences between the received signals due to the changes of structural status [47,48,50]. The energy of the stress wave transmitted from the CFRP plate to the steel beam is sensitively correlated to the bonding condition. Consequently, the energy of received signal by S2 can be used as an indicator to describe the bonding condition between the steel beam and the CFRP plate.

**Figure 4:**
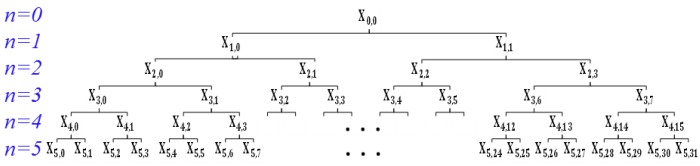
The wavelet packet decomposition of a received sensor signal

In this study, the received signal by S2 has a wide frequency range since the excitation applied to S1 is a sweep sine wave signal. The WPEI analysis is applied to decompose the received signal into a set of frequency bands, and the signal energy of each frequency band can be computed [46,50]. The total energy of the received signal can be calculated by accumulating energies of all the frequency bands. A received sensor signal X can be decomposed by *n*-level wavelet packets into $2^{n}$ frequency bands $\;\left\{X_{1},X_{2},\ldots ,X_{2^{n}}\right\}$. A higher level of decomposition leads to a more precise result, however at a higher computational cost. In this study, n=5 was selected to calculate the WPEI because it delivered an accurate enough result. The wavelet packet decomposition of a received sensor signal is shown in Figure 4.

$X_{j}$ is the decomposed signal from the original received signal X, and j represents the frequency band $\left(j=1,\;2,\ldots ,2^{n}\right)$. $X_{j}\;$ is expressed as

(1) $$X_{j}=\left[x_{j,1},x_{j,2},\ldots ,x_{j,m}\right],$$

where m  is the sampling data received by the sensor. Hence, the energy of the decomposed signal $E_{j}$ can be defined by

(2) $$E_{j}=x_{j,1}{}^{2}+x_{j,2}{}^{2}+\cdots x_{j,m}{}^{2}.$$

The gross energy E  of the received signal can be computed by the summation of all the decomposed signals. Thus, the total energy E of the signal can be expressed as

(3) $$E=\overset{2^{n}}{\underset{j=1}{\sum }}E_{j\;}.$$

The WPEI defined by Equation (3) has been applied to evaluating the structural health condition in concrete structures [56] and other applications [57,58,59,60]. In this study, the total energy of received signal by S2 can be characterized through the WPEI. The WPEI value of received signal by S2 can be used as the initial value in an area without debonding, and the value will decrease when there is debonding damage between the steel beam and the reinforcing CFRP plate.

## 3. Experimentation: Specimen, Setup, and Procedure

### 3.1. Specimen Fabrication

**Figure 5:**
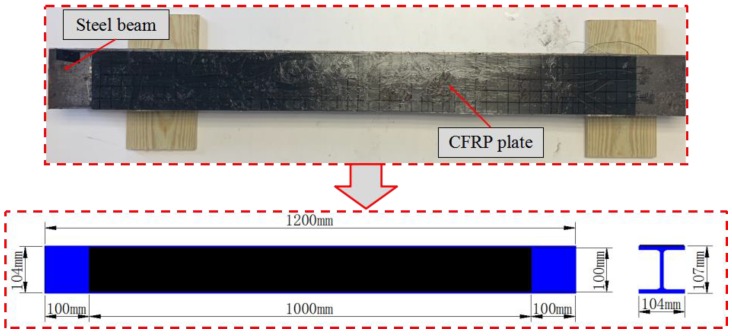
Dimensions of CFRP plate and steel beam.

The dimensions of the steel beam for the debonding detection experiment are presented in Figure 5. The dimensions of CFRP plate are 1000 mm × 100 mm × 1.5 mm. The dimensions of PZT patch and magnet are Φ 8 mm × 1 mm and Φ 15 mm × 15 mm, respectively. Table 1 shows the material properties of the transducers and the testing specimen.

**Figure 6:**
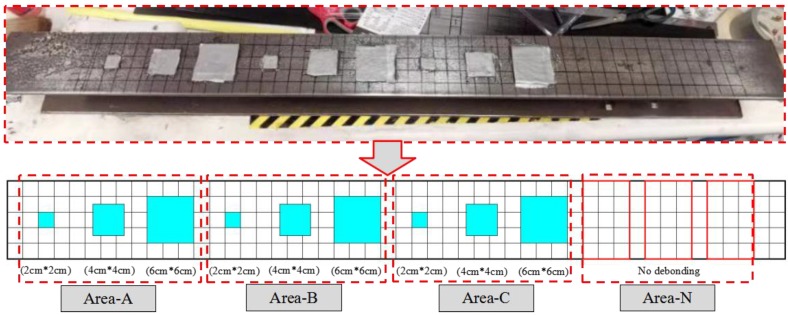
Dimensions and position of debonding damage.

The contact area of CFRP plate and steel beam was divided into 250 (5 × 50) square grids. The side of each grid was 2 cm long. In order to determine the feasibility of the debonding detection between CFRP plate and steel beam, three identical areas with debonds (Area-A, Area-B, and Area-C) were designed, and each area had three different debonding areas (2 cm × 2 cm, 4 cm × 4 cm and 6 cm × 6 cm), which were designed to determine the influence of different debonding areas on the received signal. The locations of debonding areas are shown in Figure 6. Area-N involves no debonding area and is considered as the healthy area, which can provide the baseline data. In Areas A, B, and C, the debonds were simulated by embedding the same foam material with a thickness of 2 mm between steel beam and reinforcing CFRP plate.

### 3.2. Experimental Setup and Procedure

**Figure 7:**
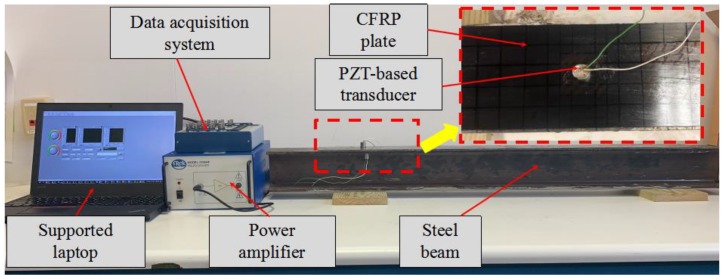
Experimental setup of the debonding detection experiment.

As shown in Figure 7, the experimental setup for the debonding detection experiment includes the steel beam, CFRP plate bonded on the steel beam, two PZT transducers (S1 and S2), a data acquisition system (NI USB6363) with 2 MS/s sampling frequency hosted by a supporting laptop, and a power amplifier with a gain of 50 for a piezoceramic load. In the process of the test, the data acquisition system generated a sweep sine wave, and the power amplifier was used to drive the PZT transducer S1 as an actuator.

**Figure 8:**
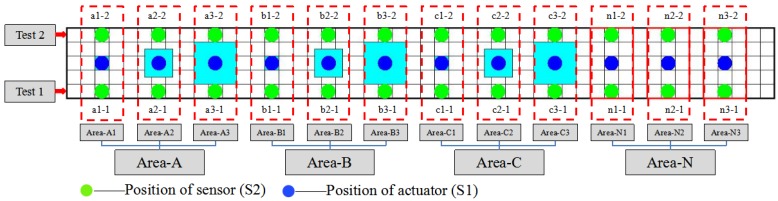
Position of actuator (S1) and sensor (S2) of each test area.

As shown in Figure 8, there are four areas (Area-A, Area-B, Area-C, and Area-N) on the steel beam/CFRP plate interface. Each area contains three test areas, which means there are 12 test areas (Area-A1, Area-A2, Area-A3, Area-B1, Area-B2, Area-B3, Area-C1, Area-C2, Area-C3, Area-N1, Area-N2, and Area-N3) in the debonding detection experiment. In order to determine the effectiveness of debonding detection, two tests (Test 1 and Test 2) were conducted in this experiment. In Test 1, the sensor (S2) was placed on the green locations whose labels were a1-1, a2-1, a3-1, b1-1, b2-1, b3-1, c1-1, c2-1, c3-1, n1-1, n2-1, and n3-1 in each test area. In Test 2, the sensor (S2) was placed on the green point on the other side of the steel beam whose labels were a1-2, a2-2, a3-2, b1-2, b2-2, b3-2, c1-2, c2-2, c3-2, n1-2, n2-2, and n3-2 in each test area. The actuator (S1) was placed at the blue point of each test area in both Test 1 and Test 2. In each test, S1, as an actuator, generated stress waves upon receiving the excitation signal, and S2, as a sensor, detected the propagating stress wave across from the epoxy layer. A sweep sine signal was used to excite the actuator S1. The frequency range, amplitude, and time interval of the sweep sine excitation signal were 1000 Hz–300 kHz, 3 V, and 0.5 s, respectively. Since all the tests used the same actuator (S1) and sensor (S2), the influence of PZT parameters on the experimental results was negligible.

## 4. Experimental Results and Analysis

**Figure 9:**
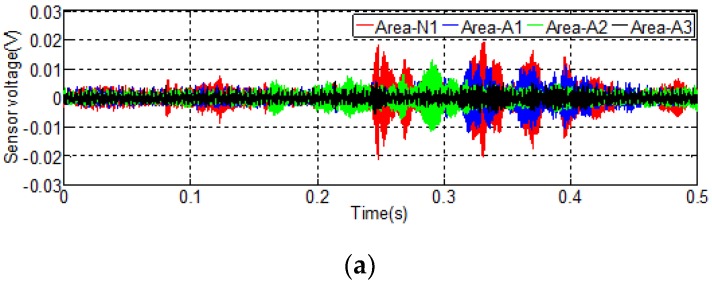

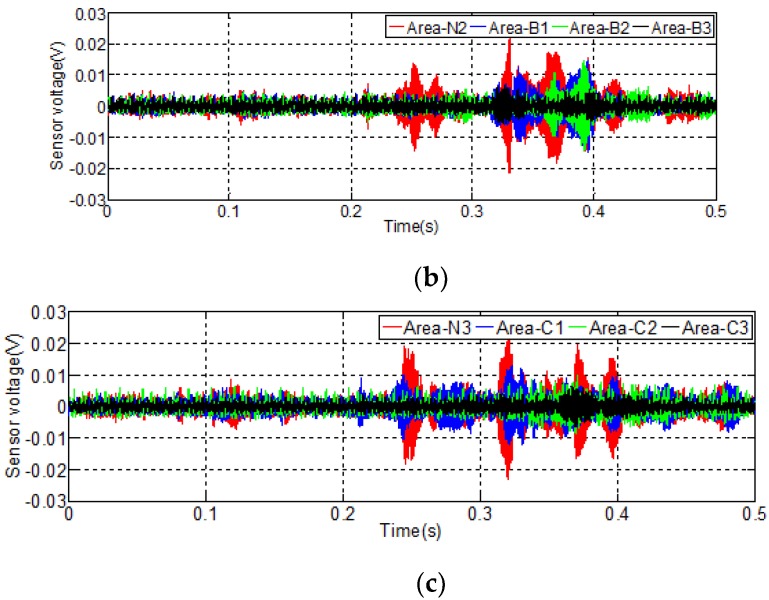
Signal received by S2 in Test 1: (**a**) signal received by S2 in Area-A and Area-N1; (**b**) signal received by S2 in Area-B and Area-N2; (**c**) signal received by S2 in Area-C and Area-N3.

The results of Test 1 in the debonding detection experiment are shown in Figure 9, where each curve represents the received signal by S2 in 0.5 s. To enable the comparison of the sensor signals without and with debonding, the received signals for the sensors in Area-N1, -N2, and -N3 are used as the baseline data and are compared to those in Area-A, -B and -C, respectively.

Figure 9a shows the time domain signal received by S2 in Areas N1, A1, A2, and A3. The aptitude of received signal in Area-N1 is between −0.02 V and 0.02 V, and it is much larger than the that of received signals in Area-A1, which reveals that the signal amplitude received by S2 in a debonding area is much smaller than that in an area without a debonding, which means the stress wave propagation from CFRP plate to steel beam is sensitive to the bonding condition. The amplitudes of the time domain signals in Area-A1 and Area-A2 have similar values; therefore, it is not possible to differentiate their difference. The WPEI analysis was applied to calculate the energy of the received signal. As the black curve shows in the figure, the signal received in Area-A3 has a much smaller amplitude than those in Area-A1 and Area-A2, which means the received signal in the debonding area of 36 cm^2^ is smaller than those in the areas of 4 cm^2^ and 9 cm^2^. Figure 9b,c shows the similar variation trend of the received signals by S2.

**Figure 10:**
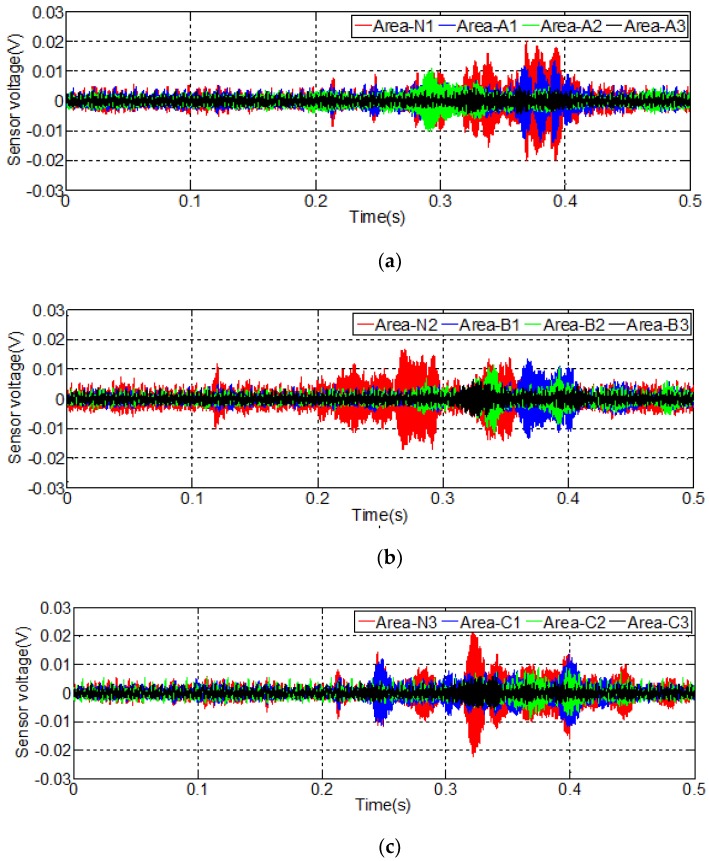
Signal received by S2 in Test 2: (**a**) signal received by S2 in Area-A and Area-N1; (**b**) signal received by S2 in Area-B and Area-N2; (**c**) signal received in S2 at Area-C and Area-N3.

The results of Test 2 in the debonding detection experiment are shown in Figure 10. By comparing the time domain signal responses in Test 2 with Test 1, a similar conclusion can be drawn.

**Figure 11:**
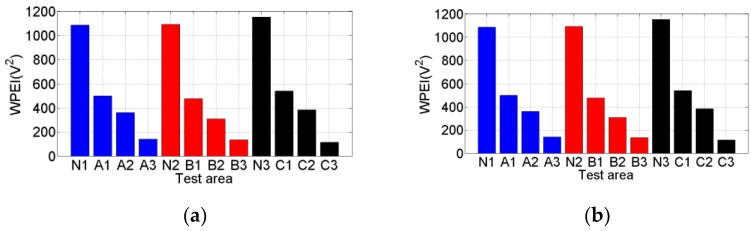
Wavelet-packet-based energy indices (WPEIs) of received signals: (**a**) WPEIs of received signals in Test 1; (**b**) WPEIs of received signals in Test 2.

**Figure 12:**
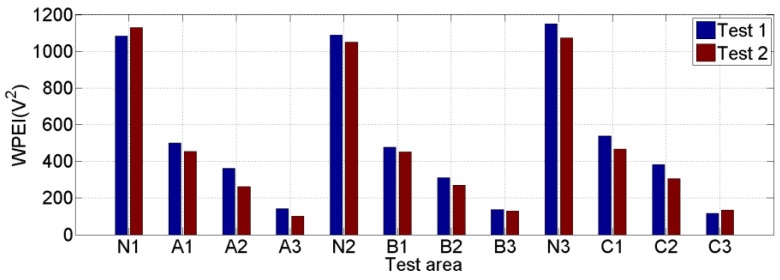
Comparison of WPEIs in Test 1 and Test 2.

In addition, the WPEI was used to quantify the total energy of the signal received by S2 in both Test 1 and Test 2, as shown in Figure 11. The Areas N1, N2, N3 have no debonding, and Area-A1, -B1, and -C1; Area-A2, -B2, and -C2; and Area-A3, -B3, and -C3 have debonding, whose areas are 4 cm^2^, 16 cm^2^, and 36 cm^2^, respectively. It can be seen in Figure 11a that the WPEIs are between 1000 and 1200 in areas without debonding, and they are much larger than the WPEIs in debonding areas. When the debonding appears (4 cm^2^), the WPEIs are reduced significantly to between 400 and 600 since the debond damage dissipates the stress wave propagation. As the debonding area increases to 16 cm^2^, the WPEIs decrease to between 200 and 400. When the debonding area further increases to 36 cm^2^, the WPEIs are much less than 200. Figure 11b shows that the trend of the WPEIs in Test 2 is almost the same as that in Test 1. Figure 12 shows the comparison of the WPEIs in Test 1 and Test 2. There is a slight difference between the amplitudes of energy indices in each area in Test 1 and Test 2 because of the different contact status between specimen and transducers. For example, the surface smoothness of steel beams in different areas can lead to a different effective contact area with the sensor. Nevertheless, the energy index trends of each test area are almost the same in both Test 1 and Test 2. The WPEIs illustrate that the PZT-based transducer can effectively detect the debonding between steel beam and reinforcing CFRP plate. Additionally, the WPEIs also reflect the dimension of the debonding: the less the WPEI value, the larger the debonding area.

## 5. Conclusions and Future Work

Carbon fiber reinforced polymer (CFRP) plate is one of the most commonly used materials for retrofitting or strengthening steel structures, and the debonding between CFRP plates and steel structure is one of the main failure modes. The debonding adversely impacts the reinforcement by reducing the effective structural load carrying capacity and even causes the sudden and brittle failure of strengthened structures. In this paper, a straightforward, removable PZT-based active sensing approach is utilized to detect the debonding between steel beam and reinforcing CFRP plate. To verify the proposed methods, two removable PZT transducers which contain PZT patch and magnet were developed and fabricated. For the specimen, there were three debonding areas and one perfect bonding area. The results of the experimental investigation show that the wavelet-packet-based energy indices (WPEIs) in three debonding areas decrease with the increase of the debonding severity. Therefore, the results demonstrate that the active sensing approach can effectively detect the debonding between a steel beam and the reinforcing CFRP plate by using removable PZT transducers. The main innovations of this paper are the development of the removable PZT transducers and the use of them in the active sensing to detect the debonding damage between steel beam and reinforcing CFRP plate. Future work will involve the integration of the proposed removable PZT transducers with robotic manipulators to automate the debonding detection of CFRP reinforced steel beams. In addition, the percussion or the tapping-and-listening-based approaches have been recently reported for damage detection [61,62,63], which motivates us to investigate detection of the debonding of the CRRP reinforcement by using the percussion approach in future work. To model the debonding process between a steel beam and the reinforcing CFRP plate, we plan to use the fractal contact theory [64,65] to analyze the stress wave attenuation across the debonding zone.

## Figures and Tables

**Table 1:** The material properties of the transducers and the test specimen.

Materials	Parameters	Value	Units
Steel beam/magnet	Density	7900	kg/m^3^
	Young’s modulus	206	Gpa
	Poisson’s ratio	0.3	/
Epoxy	Density	1250	kg/m^3^
	Young’s modulus	3.5	Gpa
	Poisson’s ratio	0.1	/
CFRP	Density	1780	kg/m^3^
	Young’s modulus	160	GPa
	Poisson’s ratio	0.05	/
PZT	Dimension	Φ 8 × 1	mm
	Piezoelectric strain coefficients (−d_31_/d_33_/d_15_)	1.75/4.00/5.90	10^−10^ C/N
